# Early, Noninvasive Clinical Indicators of Kidney Prognosis in Primary Nephrotic Syndrome: A Retrospective Exploratory Study

**DOI:** 10.1155/2022/2718810

**Published:** 2022-08-09

**Authors:** Keiji Fujimoto, Takatoshi Haraguchi, Sho Kumano, Keita Yamazaki, Nobuhiko Miyatake, Kanae Nomura, Kiyotaka Mukai, Kazuaki Okino, Norifumi Hayashi, Hiroki Adachi, Hitoshi Yokoyama, Yasuo Iida, Kengo Furuichi

**Affiliations:** ^1^Department of Nephrology, Kanazawa Medical University School of Medicine, Ishikawa 920-0293, Japan; ^2^Department of Mathematics, Kanazawa Medical University, Ishikawa 920-0293, Japan

## Abstract

This retrospective exploratory study aimed to identify early clinical indicators of kidney prognosis in primary nephrotic syndrome (NS). Univariate Cox proportional hazards regression analysis identified clinical parameters in the 2-month period after initiating immunosuppressive therapy (IST); it predicted 40% reduction in the estimated glomerular filtration rate (eGFR) in 36 patients with primary NS. Time-dependent receiver operating characteristic curve analysis was used to evaluate the performance of the predictors for the cumulative incidence of 40% reduction in the eGFR up to 8 years after initiating IST. The mean follow-up period was 71.9 months. The eGFR was reduced by 40% in four patients. Significant predictors for time to 40% reduction in the eGFR were as follows: an increase in the serum soluble urokinase plasminogen activator receptor (s-suPAR) 2 months after initiating IST (Δs-suPAR (2M); hazard ratio (HR) for every 500 pg/mL increase: 1.36, *P*=0.006), s-suPAR at 2 months after initiating IST (s-suPAR (2M); HR for every 500 pg/mL increase: 1.13, *P*=0.015), urinary protein-to-creatinine ratio (u-PCR) (u-PCR (2M); HR for every 1.0 g/gCr increase: 2.94, *P*=0.003), and urinary liver-type fatty acid-binding protein (u-L-FABP) (u-L-FABP (2M); HR for every 1.0 *μ*g/gCr increase: 1.14, *P*=0.006). All four factors exhibited high predictive accuracy for cumulative incidence of 40% reduction in the eGFR up to 8 years after initiating IST, with areas under the receiver operating characteristic curve of 0.92 for Δs-suPAR (2M), 0.87 for s-suPAR (2M), 0.93 for u-PCR (2M), and 0.93 for u-L-FABP (2M). These findings suggest that Δs-suPAR (2M), s-suPAR (2M), u-PCR (2M), and u-L-FABP (2M) could be useful indicators of initial therapeutic response for predicting kidney prognosis in primary NS.

## 1. Introduction

The status of nephrotic syndrome (NS, i.e., urinary protein concentration) at 6 months after initiating immunosuppressive therapy (IST) is an important factor for predicting kidney prognosis in NS cases [1–3]. In a large cohort study on NS in Japan, patients in whom urinary protein could not be reduced to <1 g/day even after 6 months of IST had significantly poorer kidney prognosis than those in whom urinary protein was reduced to <1 g/day. Furthermore, patients with uncontrolled urinary protein levels were at higher risk of developing end-stage kidney disease (ESKD) [3]. The Japanese Society of Nephrology defines intractable NS as NS in which urinary protein levels cannot be reduced to <1 g/day even after 6 months of IST [4]. A recent epidemiological research study in Japan found death from infectious diseases to be a critical survival prognostic factor for primary NS [5]. This finding highlights the necessity of predicting kidney prognosis before or at the earliest period after the start of initial treatment to establish individualized treatment protocols based on each patient's kidney prognosis and risk of infectious disease.

In this regard, indiscriminately administering primary treatment for 6 months only to identify intractable NS can be insufficient. In clinical practice, kidney prognosis should be predicted using valuable biomarkers at 1-2 months after commencing treatment to determine the effectiveness of each patient's management plan [4]. However, there are no validated biomarkers of treatment effectiveness within the first 2 months of IST in relation to kidney prognosis. Therefore, this study aimed to investigate the feasibility of using baseline clinical parameters to predict kidney prognosis before initiating IST. Furthermore, we aimed to determine whether changes in these clinical parameters 2 months after initiating IST can be used to predict kidney prognosis and ultimately clarify whether response to initial treatment can be predictive of kidney prognosis.

## 2. Materials and Methods

### 2.1. Study Design and Participants

In this retrospective exploratory study, we evaluated 47 patients with primary NS who were diagnosed using kidney biopsy at our hospital between January 2006 and December 2017. After excluding 11 patients who did not receive IST (all of whom had membranous nephropathy (MN)), 36 patients who received IST were included in the final analysis. Of these, 18 had minimal-change NS (MCNS), seven had focal segmental glomerulosclerosis, nine had MN, and two had membranoproliferative glomerulonephritis. The patients' clinical data were obtained from their medical records and laboratory test reports, all of which were stored in an electronic medical record system.

This study was approved by the Kanazawa Medical University Ethics Committee (No. I285) and conducted in accordance with the ethical principles outlined in the Declaration of Helsinki. All patients provided written informed consent for participation.

### 2.2. Initial IST Protocol

In 29 (80.6%) patients, the IST protocol was methylprednisolone (MP) pulse + prednisolone (PSL) + cyclosporine (CyA) and involved the administration of PSL and CyA after MP pulse (500 mg/day for 3 days). The IST protocols were PSL alone in three (8.2%), MP pulse + PSL + mizoribine in two (5.6%), and MP pulse + PSL in two (5.6%) patients.

### 2.3. Estimation of Daily Urinary Protein and Definition of Treatment Response

Daily urinary protein level was estimated as the urinary protein-to-creatinine ratio (u-PCR) in voided urine. Proteinuria remission was categorized as complete remission (CR, i.e., u-PCR <0.3 g/gCr), incomplete remission type 1 (ICR-1, i.e., u-PCR <1.0 g/gCr), and incomplete remission type 2 (ICR-2, i.e., u-PCR <3.5 g/gCr). Intractable NS was defined as NS in which u-PCR does not decrease to <1 g/gCr despite treatments, including steroids and immunosuppressors, for 6 months. These definitions were based on the NS clinical practice guidelines of the Japanese Society of Nephrology [4].

### 2.4. Calculation of Estimated Glomerular Filtration Rate (eGFR) and Measurement of the Serum and Urinary Soluble Urokinase Plasminogen Activator Receptor (suPAR) and Urinary Liver-Type Fatty Acid-Binding Protein (L-FABP)

The eGFR in adult Japanese patients aged at least 18 years was calculated using the following equation: eGFR = 194 × age (year) − 0.287 × serum creatinine (mg/dL) − 1.094 × 0.739 (if female) [6]. suPAR in sera and urine samples frozen at −80°C was measured using a commercially available enzyme-linked immunosorbent assay kit (Quantikine Human suPAR Immunoassay; R&D Systems, Minneapolis, MN, USA). L-FABP from urine samples frozen at −80°C was measured using a commercially available chemiluminescent enzyme immunoassay kit (LUMIPULSE Presto L-FABP; FUJIREBIO Inc., Hachiouji, Tokyo, Japan).

### 2.5. Statistical Analysis

For the main analysis, the time to 40% reduction in the eGFR, which is a surrogate marker of ESKD [7–10], was set as the outcome variable. Meanwhile, explanatory variables included clinical parameters at the baseline and at 2 months after initiating IST and the amount of change over 2 months (Δ). These were used in univariate Cox proportional hazards regression analysis to identify significant predictors of the time to 40% reduction in the eGFR. The following clinical parameters were examined at the baseline: age, sex, kidney tissue diagnosis (MCNS or non-MCNS), u-PCR, selectivity index, serum (s-) and urinary (u-) suPAR, u-L-FABP, eGFR, s-albumin, total cholesterol, and use of CyA in initial treatment. Two months after initiating IST, changes in the eGFR (ΔeGFR (2M)), u-PCR (Δu-PCR (2M)), u-L-FABP (Δu-L-FABP (2M)), and s- and u-suPAR (Δs-suPAR (2M), and Δu-suPAR (2M), respectively) were examined. Additional factors such as eGFR (2M), u-PCR (2M), u-L-FABP (2M), s-suPAR (2M), and u-suPAR (2M) were also evaluated. Time-dependent receiver operating characteristic (ROC) curve analysis [11, 12] using the area under the ROC curve (AUC) was performed to evaluate the predictive accuracy of the parameters for the cumulative incidence (CI) of 40% reduction in the eGFR by 2, 3, 4, 5, 6, 7, and 8 years after initiating treatment. Cutoff values were selected to maximize the sum of sensitivity and specificity.

In the secondary analysis, the outcome variable set was the achievement of ICR-1 (i.e., presence/absence of intractable NS) by 6 months after initiating IST. The same clinical parameters used in the main analysis were used as explanatory variables in univariate logistic regression analysis to identify significant predictors of intractable NS. The performance (AUC) of the predictors for intractable NS identified in the secondary analysis was evaluated using ROC curve analysis, and the cutoff values were set to maximize the sum of sensitivity and specificity.

Continuous variables are presented as means with standard deviation (SD) for normally distributed data and as the medians with interquartile range (IQR) for non-normally distributed data. All statistical analyses were performed using EZR (Saitama Medical Center, Jichi Medical University, Saitama, Japan) [13]. All *P* values were two-sided, and *P* < 0.05 was considered statistically significant. The *P* values reported in this manuscript are nominal values.

## 3. Results and Discussion

### 3.1. Baseline Characteristics

The mean patient age was 53.8 years, and the male-to-female ratio was 5 : 4. The baseline patient characteristics are summarized in Table 1. There were an equal number of patients with respect to the renal tissue type (18 patients each with MCNS and non-MCNS). Hypoalbuminemia (i.e., mean serum albumin <1.8 g/dL) due to hyperproteinemia (mean u-PCR, 10.9 g/gCr) was observed in all patients. The mean eGFR was 59.8 mL/min/1.73 m^2^. CyA was used for initial treatment in 29 (80.6%) patients.

### 3.2. Predictive Factors of Time to 40% Reduction in eGFR

**Figure 1:**
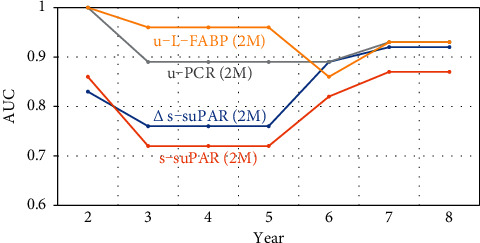
Time-dependent accuracy of each clinical parameter for predicting 40% reduction in the eGFR. suPAR, soluble urokinase receptor; s-, serum; u-, urinary; L-FABP, liver-type fatty acid-binding protein; PCR, protein-to-creatinine ratio; ROC, receiver operating characteristic; AUC, the area under the ROC curve. Predictive accuracy for the cumulative incidence of 40% reduction in the eGFR by *X* years after starting immunosuppressive therapy using time-dependent ROC curve analysis (*X* = 2, 3, 4, 5, 6, 7, and 8).

The mean follow-up period in the study cohort was 71.9 months. There were four patients (11.1%) who showed 40% reduction in the eGFR; this was achieved at 80 months in a male patient in his 70s, at 17 months in an MCNS male patient in his 80s, and at 67 months in an MN male patient in his 70s and in an MN female patient in her 50s. In the main analysis, univariate Cox proportional hazards regression analysis showed that Δs-suPAR (2M), s-suPAR (2M), u-PCR (2M), and u-L-FABP (2M) were significant predictors of 40% reduction in the eGFR (Table 2). No baseline clinical parameter showed significance.  Figure 1 shows the results of the time-dependent ROC curve analysis of the predictive accuracy (AUC) for the CI of 40% reduction in the eGFR by *X* years after initiating IST (*X* = 2, 3, 4, 5, 6, 7, and 8). The cutoff value for each parameter is shown in Table 3, and u-PCR (2M) and u-L-FABP (2M) showed stable and high predictive accuracies. The accuracies of Δs-suPAR (2M) and s-suPAR (2M) for predicting the CI of 40% reduction in the eGFR by 2–5 years after initiating IST were lower than those of the u-PCR (2M) and u-L-FABP (2M). However, the accuracies of Δs-suPAR (2M) and s-suPAR (2M) for predicting the CI of 40% reduction in the eGFR by 6–8 years after initiating IST were closer to those of the u-PCR (2M) and u-L-FABP (2M). The cutoff values of u-PCR (2M) that predict the CI of 40% reduction in the eGFR were 3.9 g/gCr (nearly ICR-2 level), 0.9 g/gCr (nearly ICR-1 level), and 0.4 g/gCr (nearly CR level) by 2, 3–5, and 6–8 years after initiating IST, respectively. The cutoff values of s-suPAR (2M) that predict the CI of 40% reduction in the eGFR ranged from 3000–4000 pg/mL at all time points.

### 3.3. Predictive Factors for Intractable NS

Using the same clinical parameters used in the main analysis, Δs-suPAR (2M), s-suPAR (2M), u-PCR (2M), and u-L-FABP (2M) were found to be significant predictors of intractable NS (secondary analysis, Table 4).  Table 5 shows the predictive accuracies (AUC) and cutoff values of these clinical parameters for intractable NS. Particularly, s-suPAR (2M) and u-PCR (2M) had high predictive accuracies (AUC). s-suPAR, which did not improve to at least 3000 pg/mL, and u-PCR, which did not improve to ICR-1 levels after 2 months of IST, were found to be predictive factors for intractable NS.

## 4. Discussion

Valuable early clinical indicators of kidney prognosis in primary NS are limited. This study found that a reduction of u-PCR (2M) values to ICR-2, ICR-1, and CR levels could suppress the CI of 40% reduction in the eGFR by 2, 3–5, and 6–8 years after initiating IST, respectively. These findings suggest that reducing u-PCR (2M) to a lower level will reduce the long-term risk of kidney function deterioration. To the best of our knowledge, this study is the first to report on the feasibility of using Δs-suPAR (2M), s-suPAR (2M), u-PCR (2M), and u-L-FABP (2M) to define initial treatment response and predict long-term kidney prognosis in primary NS.

Interestingly, this study revealed that a 3000 pg/mL cutoff value of s-suPAR (2M) predicted the occurrence of 40% reduction in the eGFR within 8 years of initiating IST, which was at the same level as the threshold of s-suPAR that induced podocyte injury in a previous study [14]. s-suPAR induces *β*3 integrin-dependent podocyte injury and is involved in the onset of proteinuria in NS [14, 15]; it has also attracted attention as a molecule involved in the onset and progression of chronic kidney disease (CKD) [16, 17]. suPAR is involved in CKD progression through the following mechanisms: it causes podocyte injury, which in turn causes glomerulosclerosis and proteinuria that lead to tubulointerstitial injury [14–17]. In addition, suPAR that crosses into the urine via glomerular filtration directly causes tubulointerstitial injury [18], which is a common pathway of CKD progression [19] that causes renal function deterioration. Therefore, increasing suPAR levels would affect tubulointerstitial injury directly and/or indirectly.

This study demonstrated that serum (s-) suPAR, not urinary (u-), predicts kidney prognosis in primary NS. We hypothesize that this is because s-suPAR is the starting point of suPAR-induced kidney injury, which makes it superior to u-suPAR (which is only involved in part of the kidney injury mechanism) as a predictor of kidney prognosis. In other words, we believe that s-suPAR levels reflect both podocyte damage caused by s-suPAR and tubulointerstitial injury caused by u-suPAR, while u-suPAR levels reflect only the latter. Further, we found that the u-PCR and u-L-FABP were superior to s-suPAR as predictors of kidney prognosis. This could be because multiple humoral factors [20–23], including s-suPAR and autoantibodies [24–26], are involved in the kidney injury observed in primary NS. This indicates that clinicians should focus on urinary protein (u-L-FABP) levels (and not on suPAR) as they directly reflect the renal damage caused by multiple humoral factors and autoantibodies, enabling more accurate predictions of kidney prognosis.

This study has several strengths. First, different statistical methods, namely, Cox proportional hazards regression analysis for 40% reduction in the eGFR [7–10] and logistic regression for intractable NS [3], identified the same predictors for the two surrogate endpoints of ESKD. This demonstrates the robustness of our results. Second, to the best of our knowledge, this study is the first to compare longitudinal changes in the performance (AUC) of suPAR, L-FABP, and u-PCR as predictors of kidney prognosis in primary NS using time-dependent ROC curve analysis. Third, the IST protocols used for initial treatment were fairly uniform, minimizing any confounders from differences in the immunosuppression protocols.

This study also has certain limitations. First, it was a single-centre study with a small sample size and heterogenous causes of NS, which limits the generalizability of the results. Second, this was an exploratory study that was mainly intended to generate hypotheses. Therefore, prospective studies with larger sample sizes are needed to verify our results.

## 5. Conclusions

In conclusion, Δs-suPAR (2M), s-suPAR (2M), u-PCR (2M), and u-L-FABP (2M) accurately predict 40% reduction in the eGFR. Thus, they may be useful noninvasive indicators for the early prediction of kidney prognosis after initial treatment in primary NS.

## Figures and Tables

**Table 1:** Baseline patient characteristics.

Characteristics	Value
Age (years, mean ± SD)	53.8 ± 20.9
Sex (n, male/female)	20/16
Kidney biopsy diagnosis (n, MCNS/non-MCNS)	18/18
u-PCR (g/gCr, mean ± SD)	10.9 ± 5.9
Selectivity index (mean ± SD)	0.19 ± 0.10
u-suPAR (pg/mgCr, mean ± SD)	3774.2 ± 1965.7
u-L-FABP (*μ*g/gCr, median (IQR))	39.5 (19.8–83.1)
eGFR (mL/min/1.73 m^2^, mean ± SD)	59.8 ± 25.0
s-Albumin (g/dL, mean ± SD)	1.8 ± 0.5
Total cholesterol (mg/dL, mean ± SD)	351.3 ± 123.0
s-suPAR (pg/mL, mean ± SD)	3488.7 ± 1641.9
Cyclosporine use in initial treatment (*n*, use/nonuse)	29/7

SD, standard deviation; MCNS, minimal-change nephrotic syndrome; s-, serum; u-, urinary; PCR, protein-to-creatinine ratio; suPAR, soluble urokinase plasminogen activator receptor; L-FABP, liver-type fatty acid-binding protein; eGFR, estimated glomerular filtration rate.

**Table 2:** Univariate Cox proportional hazard regression analysis for prediction of the time to 40% reduction in the eGFR.

Clinical parameters		Hazard ratio [95% CI]	*P* value
At baseline			
Age	Per 5 years of age	1.33 [0.93–1.91]	*0.120*
Sex	Male: 0, female: 1	0.28 [0.03–2.49]	*0.252*
Kidney tissue diagnosis	MCNS: 0, non-MCNS: 1	2.18 [0.23–20.37]	*0.495*
u-PCR	Per 1.0 g/gCr	0.96 [0.80–1.16]	*0.685*
Selectivity index	<0.2 : 0, ≥0.2 : 1	1.81 [0.30–10.91]	*0.516*
u-suPAR	Per 500 pg/mgCr	1.04 [0.82–1.33]	*0.732*
u-L-FABP	Per 1.0 *μ*g/gCr	1.00 [1.00–1.01]	*0.718*
eGFR	Per 10 mL/min/1.73 m^2^	0.86 [0.60–1.24]	*0.425*
s-Albumin	Per 1.0 g/dL	3.39 [0.43–27.02]	*0.248*
Total cholesterol	Per 10 mg/dL	0.92 [0.81–1.05]	*0.214*
s-suPAR	Per 500 pg/mL	1.17 [0.98–1.40]	*0.081*
Cyclosporine use in initial treatment	Nonuse: 0, use: 1	0.29 [0.05–1.83]	*0.189*

Change (Δ) during 2 months after initiating IST			
Δu-PCR (2M)	Per 1.0 g/gCr	1.16 [0.91–1.47]	*0.227*
Δu-suPAR (2M)	Per 500 pg/mgCr	1.34 [0.92–1.97]	*0.130*
Δu-L-FABP (2M)	Per 1.0 *μ*g/gCr	1.00 [0.99–1.01]	*0.994*
ΔeGFR (2M)	Per 10 mL/min/1.73 m^2^	0.82 [0.44–1.53]	*0.523*
Δs-suPAR (2M)	Per 500 pg/mL	1.36 [1.09–1.69]	*0.006*

At 2 months after initiating IST			
u-PCR (2M)	Per 1.0 g/gCr	2.94 [1.43–6.03]	*0.003*
u-suPAR (2M)	Per 500 pg/mgCr	1.20 [1.00–1.44]	*0.050*
u-L-FABP (2M)	Per 1.0 *μ*g/gCr	1.14 [1.04–1.26]	*0.006*
eGFR (2M)	Per 10 mL/min/1.73 m^2^	0.79 [0.54–1.15]	*0.215*
s-suPAR (2M)	Per 500 pg/mL	1.13 [1.03–1.23]	*0.015*

MCNS, minimal-change nephrotic syndrome; s-, serum; u-, urinary; PCR, protein-to-creatinine ratio; suPAR, soluble urokinase plasminogen activator receptor; L-FABP, liver-type fatty acid-binding protein; eGFR, estimated glomerular filtration rate; IST, immunosuppressive therapy; 95% CI, confidence interval.

**Table 3:** Optimal cutoff values of predictive parameters for the CI of 40% reduction in the eGFR by *X* years after the initiation of IST.

Clinical parameters	*X* = 2 (sensitivity, specificity)	*X* = 3 (sensitivity, specificity)	*X* = 4 (sensitivity, specificity)	*X* = 5 (sensitivity, specificity)	*X* = 6 (sensitivity, specificity)	*X* = 7 (sensitivity, specificity)	*X* = 8 (sensitivity, specificity)
ΔS-suPAR (2M; pg/mL)	686.6 (1.00, 0.83)	225.6 (0.97, 0.74)	225.6 (0.97, 0.74)	225.6 (0.97, 0.74)	225.6 (0.92, 0.80)	3.4 (0.95, 0.79)	3.4 (0.95, 0.79)
u-PCR (2M; g/gCr)	3.9 (1.00, 1.00)	0.9 (0.97, 0.79)	0.9 (0.97, 0.79)	0.9 (0.97, 0.79)	0.4 (0.92, 0.80)	0.4 (0.93, 0.86)	0.4 (0.93, 0.86)
u-L-FABP (2M; *μ*g/gCr)	27.0 (1.00, 1.00)	11.3 (0.97, 0.91)	11.3 (0.97, 0.91)	11.3 (0.97, 0.91)	4.8 (0.97, 0.68)	10.0 (0.93, 1.00)	10.0 (0.93, 1.00)
s-suPAR (2M; pg/mL)	4222.1 (1.00, 0.86)	2836.4 (0.97, 0.59)	2836.4 (0.97, 0.59)	2836.4 (0.97, 0.59)	3978.2 (0.83, 0.89)	3501.5 (0.88, 0.92)	3501.5 (0.88, 0.92)

Δs-suPAR (2M), change (Δ) in the serum soluble urokinase plasminogen activator receptor (suPAR) within 2 months of initiating immunosuppressive therapy (IST); u-PCR (2M), urinary protein-to-creatinine ratio at 2 months after initiating IST; u-L-FABP (2M), urinary liver-type fatty acid-binding protein at 2 months after initiating IST; s-suPAR (2M), serum suPAR at 2 months after initiating IST; eGFR, estimated glomerular filtration rate.

**Table 4:** Univariate logistic regression analysis of predictive factors for intractable NS.

Clinical parameters		Odds ratio [95% CI]	*P* value
At baseline			
Age	Per 5 years of age	1.24 [0.95–1.62]	*0.111*
Sex	Male: 0, female: 1	0.92 [0.17–4.89]	*0.925*
Kidney tissue diagnosis	MCNS: 0, non-MCNS: 1	8.50 [0.90–80.03]	*0.061*
u-PCR	Per 1.0 g/gCr	0.91 [0.75–1.10]	*0.327*
Selectivity index	<0.2 : 0, ≥0.2 : 1	4.09 [0.67–24.83]	*0.126*
u-suPAR	Per 500 pg/mgCr	0.94 [0.75–1.19]	*0.628*
u-L-FABP	Per 1.0 *μ*g/gCr	1.00 [0.99–1.01]	*0.944*
eGFR	Per 10 mL/min/1.73 m^2^	0.87 [0.62–1.22]	*0.422*
s-Albumin	Per 1.0 g/dL	1.61 [0.26–9.90]	*0.610*
Total cholesterol	Per 10 mg/dL	0.95 [0.86–1.05]	*0.286*
s-suPAR	Per 500 pg/mL	1.16 [0.93–1.44]	*0.199*
Cyclosporine use in initial treatment	Nonuse: 0, use: 1	0.21 [0.03–1.33]	*0.098*

Change (Δ) within the first 2 months of IST			
Δu-PCR (2M)	Per 1.0 g/gCr	1.32 [0.99–1.77]	*0.063*
Δu-suPAR (2M)	Per 500 pg/mgCr	1.36 [0.95–1.93]	*0.093*
Δu-L-FABP (2M)	Per 1.0 *μ*g/gCr	1.00 [0.99–1.01]	*0.809*
ΔeGFR (2M)	Per 10 mL/min/1.73 m^2^	0.77 [0.43–1.40]	*0.397*
Δs-suPAR (2M)	Per 500 pg/mL	2.40 [1.15–5.03]	*0.020*

At 2 months after initiating IST			
u-PCR (2M)	Per 1.0 g/gCr	7.87 [2.02–30.73]	*0.003*
u-suPAR (2M)	Per 500 pg/mgCr	1.14 [0.92–1.42]	*0.238*
u-L-FABP (2M)	Per 1.0 *μ*g/gCr	1.14 [1.01–1.29]	*0.038*
eGFR (2M)	Per 10 mL/min/1.73 m^2^	0.74 [0.50–1.11]	*0.149*
s-suPAR (2M)	Per 500 pg/mL	1.44 [1.01–2.04]	*0.041*

MCNS, minimal-change nephrotic syndrome; s-, serum; u-, urinary; PCR, protein-to-creatinine ratio; suPAR, soluble urokinase plasminogen activator receptor; L-FABP, liver-type fatty acid-binding protein; eGFR, estimated glomerular filtration rate; IST, immunosuppressive therapy.

**Table 5:** Cutoff values of predictive factors for intractable NS.

Clinical parameters	AUC [95% CI]	Cutoff value (sensitivity, specificity)
Δs-suPAR (2M; pg/mL)	0.84 [0.65–1.00]	93.5 (0.76, 0.86)
u-PCR (2M; g/gCr)	0.98 [0.93–1.00]	1.1 (0.97, 1.00)
u-L-FABP (2M; *μ*g/gCr)	0.78 [0.59–0.96]	4.1 (0.59, 1.00)
s-suPAR (2M; pg/mL)	0.85 [0.72–0.99]	3297.0 (0.79, 0.86)

Δs-suPAR (2M), change (Δ) in the serum soluble urokinase plasminogen activator receptor (suPAR) within the first 2 months of immunosuppressive therapy (IST); u-PCR (2M), urinary protein-to-creatinine ratio at 2 months after initiating IST; u-L-FABP (2M), urinary liver-type fatty acid-binding protein at 2 months after initiating IST; s-suPAR (2M), serum suPAR at 2 months after initiating IST; AUC, the area under the ROC curve; 95% CI, 95% confidence interval.

## Data Availability

The data used to support the findings of this study are available from the corresponding author upon request.

## References

[1] Cattran D. C., Pei Y., Greenwood C. M., Ponticelli C., Passerini P., Honkanen E. (1997). Validation of a predictive model of idiopathic membranous nephropathy: its clinical and research implications. *Kidney International*.

[2] Matalon A., Valeri A., Appel G. B. (2000). Treatment of focal segmental glomerulosclerosis. *Seminars in Nephrology*.

[3] Shiiki H., Saito T., Nishitani Y. (2004). Prognosis and risk factors for idiopathic membranous nephropathy with nephrotic syndrome in Japan. *Kidney International*.

[4] Nishi S., Ubara Y., Utsunomiya Y. (2016). Evidence-based clinical practice guidelines for nephrotic syndrome 2014. *Clinical and Experimental Nephrology*.

[5] Yamamoto R., Imai E., Maruyama S. (2020). Incidence of remission and relapse of proteinuria, end-stage kidney disease, mortality, and major outcomes in primary nephrotic syndrome: the Japan nephrotic syndrome cohort study (JNSCS). *Clinical and Experimental Nephrology*.

[6] Matsuo S., Imai E., Horio M. (2009). Revised equations for estimated GFR from serum creatinine in Japan. *American Journal of Kidney Diseases*.

[7] Levey A. S., Inker L. A., Matsushita K. (2014). GFR decline as an end point for clinical trials in CKD: a scientific workshop sponsored by the national kidney foundation and the US food and drug administration. *American Journal of Kidney Diseases*.

[8] Lambers Heerspink H. J., Tighiouart H., Sang Y. (2014). GFR decline and subsequent risk of established kidney outcomes: a meta-analysis of 37 randomized controlled trials. *American Journal of Kidney Diseases*.

[9] Mol P. G., Maciulaitis R., Vetter T. (2014). GFR decline as an end point for clinical trials in CKD: a view from Europe. *American Journal of Kidney Diseases*.

[10] Thompson A., Lawrence J., Stockbridge N. (2014). GFR decline as an end point in trials of CKD: a viewpoint from the FDA. *American Journal of Kidney Diseases*.

[11] Heagerty P. J., Zheng Y. (2005). Survival model predictive accuracy and ROC curves. *Biometrics*.

[12] Heagerty P. J., Lumley T., Pepe M. S. (2000). Time-dependent ROC curves for censored survival data and a diagnostic marker. *Biometrics*.

[13] Kanda Y. (2013). Investigation of the freely available easy-to-use software ‘EZR’ for medical statistics. *Bone Marrow Transplantation*.

[14] Wei C., El Hindi S., Li J. (2011). Circulating urokinase receptor as a cause of focal segmental glomerulosclerosis. *Nature Medicine*.

[15] Yoo T. H., Pedigo C. E., Guzman J. (2015). Sphingomyelinase-like phosphodiesterase 3b expression levels determine podocyte injury phenotypes in glomerular disease. *Journal of the American Society of Nephrology*.

[16] Zeier M., Reiser J. (2017). suPAR and chronic kidney disease-a podocyte story. *Pfluegers Archiv European Journal of Physiology*.

[17] Hayek S. S., Sever S., Ko Y. A. (2015). Soluble urokinase receptor and chronic kidney disease. *New England Journal of Medicine*.

[18] Han R., Hu S., Qin W. (2019). C3a and suPAR drive versican V1 expression in tubular cells of focal segmental glomerulosclerosis. *JCI Insight*.

[19] Nangaku M. (2006). Chronic hypoxia and tubulointerstitial injury: a final common pathway to end-stage renal failure. *Journal of the American Society of Nephrology*.

[20] McCarthy E. T., Sharma M., Savin V. J. (2010). Circulating permeability factors in idiopathic nephrotic syndrome and focal segmental glomerulosclerosis. *Clinical Journal of the American Society of Nephrology*.

[21] Cheung P. K., Klok P. A., Baller J. F., Bakker W. W. (2000). Induction of experimental proteinuria in vivo following infusion of human plasma hemopexin. *Kidney International*.

[22] Cheung W., Wei C. L., Seah C. C., Jordan S. C., Yap H. K. (2004). Atopy, serum IgE, and interleukin-13 in steroid–responsive nephrotic syndrome. *Pediatric Nephrology*.

[23] Lai K. W., Wei C. L., Tan L. K. (2007). Overexpression of interleukin-13 induces minimal-change-like nephropathy in rats. *Journal of the American Society of Nephrology*.

[24] Beck L. H., Bonegio R. G., Lambeau G. (2009). M-type phospholipase A2 receptor as target antigen in idiopathic membranous nephropathy. *New England Journal of Medicine*.

[25] Tomas N. M., Beck L. H., Meyer-Schwesinger C. (2014). Thrombospondin type-1 domain-containing 7A in idiopathic membranous nephropathy. *New England Journal of Medicine*.

[26] Vallota E. H., Forristal J., Spitzer R. E., Davis N. C., West C. D. (1971). Continuing C3 breakdown after bilateral nephrectomy in patients with membrano-proliferative glomerulonephritis. *Journal of Clinical Investigation*.

